# Prevention of postoperative nausea and vomiting after orthognathic surgery: a scoping review

**DOI:** 10.1186/s12871-024-02510-z

**Published:** 2024-03-28

**Authors:** Peng-fei Gao, Le Zhao, Shi-yue Li, Yue Li, Ming-kai Chen, Jing Fu, Yang Ji

**Affiliations:** State Key Laboratory of Oral Diseases, Department of Anesthesiology, West China Hospital of Stomatology, National Clinical Research Center for Oral Diseases, Sichuan University, Chengdu, 610041 China

**Keywords:** Postoperative nausea and vomiting, Orthognathic surgery, Anesthesia, Antiemetics

## Abstract

**Introduction:**

Postoperative nausea and vomiting (PONV) is one of the most common adverse events following orthognathic surgery. It’s a distressing feeling for patients and continues to be the cause of postoperative complications such as bleeding, delayed healing, and wound infection. This scoping review aims to identify effective PONV prophylaxis strategies during orthognathic surgery that have emerged in the past 15 years.

**Methods:**

We searched Pubmed, Cochrane Controlled Register of Trials, and Embase from 2008 to May 2023. Studies meeting the following criteria were eligible for inclusion: (1) recruited patients undergo any orthognathic surgery; (2) evaluated any pharmacologic or non-pharmacologic method to prevent PONV. Studies meeting the following criteria were excluded: (1) case series, review papers, or retrospective studies; (2) did not report our prespecified outcomes.

**Results:**

Twenty-one studies were included in this review. Pharmacological methods for PONV prevention include ondansetron and dexamethasone (3 studies), peripheral nerve block technique (4 studies), dexmedetomidine (1 study), pregabalin (2 studies), nefopam (2 studies), remifentanil (1 study), propofol (2 studies), and penehyclidine (1 study). Non-pharmacologic methods include capsicum plaster (1 study), throat packs (2 studies) and gastric aspiration (2 studies).

**Conclusions:**

Based on current evidence, we conclude that prophylactic antiemetics like dexamethasone, ondansetron, and penehyclidine are the first defense against PONV. Multimodal analgesia with nerve block techniques and non-opioid analgesics should be considered due to their notable opioid-sparing and PONV preventive effect. For the non-pharmacological methods, throat packs are not recommended for routine use because of their poor effect and serious complications. More prospective RCTs are required to confirm whether gastric aspiration can prevent PONV effectively for patients undergoing orthognathic surgery.

## Introduction

Postoperative nausea and vomiting (PONV) is one of the most common adverse events following orthognathic surgery. The incidence of PONV is approximately 30% in the general surgical population [1]. In contrast, patients undergoing orthognathic surgeries suffer much higher risks of PONV, 59.4% of them experienced postoperative nausea (PON) and 28.4% experienced postoperative vomiting (POV) [2]. Frequent PONV is a distressing experience even worse than postoperative pain [3]. It can result in prolonged hospital stay and increased risk of postoperative complications such as bleeding, delayed healing, and wound infection [4].

The etiology of PONV after orthognathic surgery is multifactorial, including patient, surgical, and anesthesia factors. Female patients, less than 25 years old, bimaxillary surgery, procedures more than 3 h, and receiving more than 25 ml/kg intravenous fluids have all been implicated as causative factors in PONV [5–7]. Although antiemetics such as 5-HT_3_ receptor antagonists and dexamethasone are routinely used in orthognathic surgery, their effects on PONV prevention are limited [8, 9].

In recent years, several clinical trials have been conducted by anesthesiologists and surgeons to assess the efficacy of different methods to prevent PONV. Nevertheless, the results of these studies on this topic are contradictory, and not all methods worked well. Up to now, no relevant review or guideline has been summarized for PONV prophylaxis following orthognathic surgery. Thus, this scoping review aims to identify effective PONV prophylaxis strategies during orthognathic surgery that have emerged in the past 15 years.

## Materials and methods

This scoping review was conducted and reported according to the Preferred Reporting Items for Systematic reviews and Meta-Analyses extension for Scoping Reviews (PRISMA-ScR).

### Search strategy

Two authors independently searched Pubmed, Cochrane Controlled Register of Trials, and Embase from 2008 to May 2023. To avoid the omission of relevant studies, we selected the “All Fields” option rather than “Title/Abstract.” The search strategy was constructed using a combination of the following words: (orthognathic surgery OR bimaxillary osteotomy OR jaw surgery OR mandibular osteotomy) AND (nausea OR vomiting OR emesis). There was no language restriction during the electronic searches.

### Inclusion and exclusion criteria

Studies meeting the following criteria were eligible for inclusion: (1) recruited patients undergo any orthognathic surgery; (2) evaluated any pharmacologic or non-pharmacologic method to prevent PONV. Studies meeting the following criteria were excluded: (1) case series, review papers, or retrospective studies; (2) did not report our prespecified outcomes.

### Data extraction

Data extraction was performed independently by two authors using a prespecified data extraction form designed by PFG. Disagreements between reviewers were resolved by discussion with a third reviewer. The following information was extracted from the eligible articles: primary author, publication year, type of surgery, intervention methods, number of patients, the occurrence of postoperative nausea and/or vomiting and its *P* value.

## Results

### Study selection

**Fig. 1:**
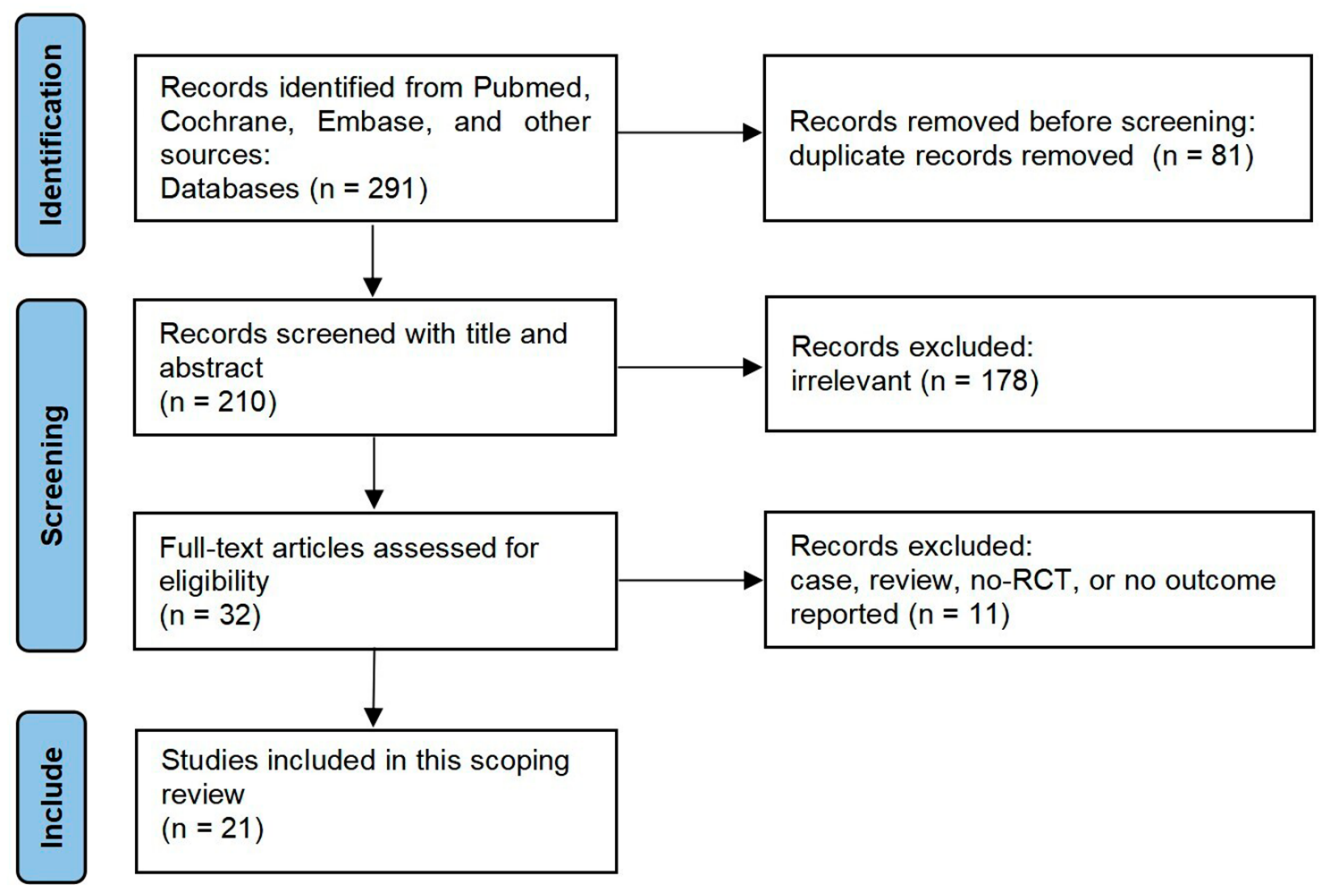
Flowchart of study selection process

A flow diagram summarized the detailed steps of our study selection was described in Fig. 1. Our initial search yielded 291 studies from Pubmed, Cochrane Controlled Register of Trials, and Embase. 210 studies remained after adjusting for duplicates. After screening the titles and abstracts, 178 studies were determined to be not relevant to this scoping review. After screening the full text, 11 studies were excluded according to our exclusion criteria. Finally, 21 RCTs were included in this review [9–29].

### Study characteristics

The characteristics of included studies are presented in Table 1. All of the twenty-one enrolled RCTs were single-center randomized controlled trials published between 2009 and 2023. Pharmacological methods for PONV prevention include ondansetron and dexamethasone (3 studies), peripheral nerve block technique (4 studies), dexmedetomidine (1 study), pregabalin (2 studies), nefopam (2 studies), remifentanil (1 study), propofol (2 studies), and penehyclidine (1 study). Non-pharmacological methods include capsicum plaster (1 study), throat packs (2 studies) and gastric aspiration (2 studies).

**Table 1:** Characteristics of PONV Prophylaxis Following Orthognathic Studies

Author (Year)	Procedure	Intervention	Intervention/Control	Intervention/ Control	Time of Intervention	Efficacy Outcome
Samieirad (2018)	orthognathic surgery	pharmacological	ondansetron + dexamethasone vs. clonidine + dexamethasone	15/15	intraoperative	PON: 53.3% vs. 73.3% at 24 h POV: 0% vs. 6.7% at 24 h
Gecaj-Gashi (2012)	orthognathic surgery	pharmacological	dexamethasone vs. metoclopramide	11/11	preoperative	PON: 9.0% vs. 27.2% at 6 h * POV: 0% vs. 18.1% at 6 h *
Lin (2017)	orthognathic surgery	pharmacological	dexamethasone 15 mg vs. dexamethasone 5 mg	31/25	preoperative	PON: 9.7% vs. 8.0% in inpatient hospital stay POV: 12.9% vs. 16.0% in inpatient hospital stay
Chatellier (2012)	mandibular surgery	pharmacological	bilateral inferior alveolar nerve block vs. placebo	16/14	preoperative	PONV: 6.3% vs. 42.9% at 24 h *
Vetter (2020)	mandibular surgery	pharmacological	bilateral inferior alveolar nerve block vs. placebo	26/25	preoperative	PONV: 15.4% vs. 40% at 24 h *
Wang (2021)	bimaxillary surgery	pharmacological	bilateral trigeminal nerve block vs. placebo	20/20	preoperative	POV: 20% vs. 40% at 24 h *
Bertuit (2021)	mandibular surgery	pharmacological	bilateral mandibular nerve block vs. placebo	50/57	preoperative	PONV: 46.0% vs. 21.1% at 24 h *
Labafchi (2023)	bimaxillary surgery	pharmacological	dexmedetomidine vs. placebo	30/30	perioperative	PON: 3.3% vs. 46.7% at 24 h *
Ahiskalioglu (2016)	double jaw surgery	pharmacological	pregabalin vs. placebo	20/20	preoperative	PONV: 10% vs. 40% at 24 h *
Khajavi (2018)	orthognathic surgery	pharmacological	pregabalin + clonidine vs. placebo	35/35	preoperative	PONV: 5.7% vs. 14.7% at 3 h *
Park (2016)	bimaxillary osteotomy	pharmacological	nefopam vs. placebo	20/21	perioperative	PON: 15% vs. 33% at 24 h POV: 15% vs. 29% at 24 h
Choi (2019)	bimaxillary surgery	pharmacological	nefopam vs. fentanyl	48/41	postoperative	PONV: 27.7% vs. 17.1% at 8 h
Nooh (2013)	Le Fort I osteotomy	pharmacological	remifentanil vs. fentanyl	8/9	intraoperative	PON: 50% vs. 55.6% in PACU POV: 25% vs. 33.3% in PACU
Tabrizi (2012)	bimaxillary surgery	pharmacological	propofol vs. isoflurane	32/30	intraoperative	PONV: 3.1% vs. 16.7% in PACU
Lin (2016)	bimaxillary surgery	pharmacological	propofol vs. sevoflurane switch to propofol vs. sevoflurane	21/21/21	intraoperative	PONV: 9.5% vs. 14.3% vs. 28.6% in inpatient hospital stay
Wang (2021)	bimaxillary surgery	pharmacological	bolus penehyclidine vs. bolus plus continuous penehyclidine vs. placebo	117/118/118	perioperative	PONV: 40.2% vs. 28.0% vs. 61.0% at 72 h *
Kim (2009)	mandibular surgery	non-pharmacological	capsicum plaster at Hegu acupoints vs. capsicum plaster on the shoulders vs. placebo	28/28/28	preoperative	PON: 10.7% vs. 42.9% vs. 46.4% at 72 h * POV: 7.1% vs. 39.3% vs. 42.9% at 72 h *
Faro (2020)	orthognathic surgery	non-pharmacological	throat packs vs. placebo	25/25	intraoperative	PON: 32% vs. 36% at 24 h POV: 24% vs. 24% at 24 h
Powell (2021)	orthognathic surgery	non-pharmacological	throat packs vs. placebo	15/15	intraoperative	PONV: 26.7% vs. 26.7% at 24 h
Schmitt (2016)	orthognathic surgery	non-pharmacological	gastric aspiration vs. placebo	12/12	postoperative	PONV: 33.3% vs. 33.3% in inpatient hospital stay
Oliveira (2022)	bimaxillary surgery	non-pharmacological	gastric aspiration vs. placebo	39/44	postoperative	POV: 15.4% vs. 36.4% at 24 h *

PONV = Postoperative Nausea and Vomiting; PON = Postoperative Nausea; POV = Postoperative Vomiting; PACU = Postanesthesia Care Unit; * = Reach Statistical Difference

### Pharmacological methods

#### Ondansetron and dexamethasone

Three RCTs investigated ondansetron or dexamethasone. Samieirad et al. [9] compared the preventive antiemetic effects of oral ondansetron combined with dexamethasone versus clonidine combined with dexamethasone administered 1 h before surgery. The incidence of PON or POV did not reach statistical difference between the two groups (53.3% vs. 73.3%, *P* = 0.256; 0% vs. 6.7%, *P* = 1.00). Gecaj-Gashi et al. [10] reported that dexamethasone administered before induction of anesthesia was more effective in preventing PON and POV than metoclopramide during 6 h postoperative (9.0% vs. 27.2%, *P* < 0.05; 0% vs. 18.1%, *P* < 0.05). However, a study by Lin et al. [11] found that a higher dose of dexamethasone (15 mg vs. 5 mg) did not further reduce the incidence of PON (9.7% vs. 8.0%, *P* = 0.90) or POV (12.9% vs. 16%, *P* = 0.74).

#### Peripheral nerve block technique

Four RCTs assessed peripheral nerve block in orthognathic surgery. Both Chatellier et al. [12] and Vetter et al. [13] found that bilateral inferior alveolar nerve block significantly decreased the incidence of PONV during the first 24 h after mandibular osteotomy (6.3% vs. 42.9%, *P* = 0.031; 15.4% vs. 40%, *P* = 0.049). For patients undergoing bimaxillary surgery, ultrasound-guided trigeminal nerve block with 0.25% ropivacaine was effective in reducing intraoperative sufentanil and remifentanil consumption and decreasing the incidence of POV (20% vs. 40%, *P* = 0.028) [14]. A study by Bertuit et al. [15] examined the role of bilateral mandibular block with 0.75% ropivacaine. Their results suggested that bilateral mandibular block effectively decreased morphine consumption but resulted in more incidence of PONV (46.0% vs. 21.1%, *P* < 0.01).

#### Dexmedetomidine

One RCT compared dexmedetomidine and placebo in patients undergoing bimaxillary surgery [16]. This study revealed that dexmedetomidine significantly reduced the incidence of PON (3.3% vs. 47.6%, *P* < 0.001) and postoperative pain scores in the first 24 h after surgery.

#### Pregabalin

Two RCTs investigated preoperative oral pregabalin for postoperative pain control and PONV risk. During double jaw surgery, Ahiskalioglu et al. [17] concluded that preoperative oral 150 mg pregabalin significantly reduced postoperative opioid consumption as well as pain scores. The incidence of PONV was lower in the pregabalin group (10% vs. 40%, *P* = 0.028). Similarly, Khajavi et al. [18] determined that perioperative oral 300 mg pregabalin and 0.2 mg clonidine can improve postoperative pain control in the early stages of recovery. The amount of opioids and incidence of PONV were also lower in patients who received pregabalin and clonidine (5.7% vs. 14.7%, *P* = 0.005).

#### Nefopam

Two RCTs comparing nefopam to placebo or fentanyl for PONV prophylaxis were identified. Park et al. [19] investigated the analgesic efficacy and side effects of nefopam. They found that nefopam was an effective analgesic in bimaxillary osteotomy but had no statistical influence on PON (15% vs. 33%, *P >* 0.05) or POV (15% vs. 29%, *P >* 0.05). Another prospective randomized controlled trial compared 120 mg nefopam with 700 µg fentanyl used in patient-controlled analgesia (PCA) following bimaxillary orthognathic surgery [20]. Similarly, no difference of PONV was observed between nefopam and fentanyl (27.7% vs. 17.1%, *P* = 0.568).

#### Remifentanil

One RCT compared the administration of remifentanil and fentanyl in patients undergoing Le Fort I osteotomy [21]. This study revealed that remifentanil and fentanyl resulted in similar incidence of PON (50% vs. 55.6%, *P >* 0.05) and POV (25% vs. 33.3%, *P >* 0.05) in postanesthesia care unit (PACU).

#### Propofol

Two RCTs compared propofol with inhalational anesthetics for PONV prevention in orthognathic surgeries. Tabrizi et al. [22] determined that propofol and isoflurane used for anesthesia maintenance resulted in a similar incidence of PONV after bimaxillary orthognathic surgery (3.1% vs. 16.7%, *P >* 0.05). Lin et al. [23] designed a prospective study to compare three anesthesia protocols (including propofol alone, sevoflurane switch to propofol, and sevoflurane alone) to compare the incidence of PONV. Patients in the propofol group had the lowest PONV rate, but it did not reach statistical difference (9.5% vs. 14.3% vs. 28.6%, *P* = 0.343).

#### Penehyclidine

One RCT with 3 groups compared the administration of penehyclidine (0.5 mg bolus), penehyclidine (0.25 mg bolus plus 0.25 mg in PCA), and placebo in patients undergoing bimaxillary surgery [24]. This study concluded that both single bolus and single bolus plus a continuous infusion of penehyclidine were effective in preventing PONV (40.2% vs. 28.0% vs. 61.0%, *P* < 0.003).

### Non-pharmacological methods

#### Capsicum plaster

One prospective RCT with 3 groups (Hegu group = capsicum plaster at the Hegu acupoints, sham group = capsicum plaster on the shoulders, and control group) demonstrated that capsicum plaster at the Hegu acupoints decreased PON (10.7% vs. 42.9% vs. 46.4%, *P* < 0.01), POV (7.1% vs. 39.3% vs. 42.9%, *P* < 0.01), and opioid requirement after orthognathic surgery [25]. Patients in the Hegu group had significantly lower pain intensity (*P* < 0.001).

#### Throat packs

Two RCTs assessed throat packs versus placebo for PONV prophylaxis. A study by Faro et al. [26] concluded that throat packs can not prevent PON (32% vs. 36%, *P* = 0.765) or POV (24% vs. 24%, *P* = 1.000) but were resulted in worse sore throat (72% vs. 36%, *P* = 0.011) and postoperative dysphagia (60% vs. 12%, *P* < 0.001). Similarly, Powell et al. [27] showed that there was no difference in the incidence of PONV when patients received throat packs (26.7% vs. 26.7%, *P* = 1.000).

#### Gastric aspiration

Two RCTs explored the effect of gastric aspiration by a gastric tube before tracheal extubation to decrease PONV. Schmitt et al. [28] evaluated gastric aspiration in orthognathic surgical patients. Their study found no difference in the overall incidence of PONV (33.3% vs. 33.3%, *P* = 1.000). Another similar study by Oliveira et al. [29] analyzed 83 patients. Nevertheless, their result showed that gastric aspiration can reduce the risk of postoperative vomiting (15.4% vs. 36.4%, *P* = 0.031).

## Discussion

Opioids have been the cornerstone of perioperative analgesia but are also the main factor causing PONV. In recent years, regional anesthesia techniques and various non-opioid analgesic medications have been promoted to decrease opioids consumption and their side effects. In orthognathic surgery, the maxillary and mandibular branches of the trigeminal nerve can be blocked prior to surgery [30]. Peripheral nerve block techniques provide preemptive analgesia and prevent central sensitization, thereby reducing the surgical stress response and alleviating postoperative pain [31].

In this review, most studies revealed significantly lower incidences of PONV when combined nerve block with general anesthesia. However, Bertuit et al. [15] reported conflicting findings. They found bilateral mandibular block was associated with a higher incidence of PONV, although postoperative morphine consumption was reduced. The author explained that the higher incidence of PONV may be due to the higher Apfel score in the block group, which is the most widely used tool for risk stratification of PONV [1].

Non-opioid medications such as dexmedetomidine and pregabalin also have beneficial effect on PONV prevention. Dexmedetomidine is a highly selective α-2 adrenergic receptor agonist and possesses analgesic, anxiolytic, sympatholytic effects. Recent clinical trials revealed that dexmedetomidine can reduce the incidence of PONV after dental rehabilitation and thoracoscopic lung cancer resection [32, 33]. Pregabalin is a structural gama-aminobutyric acid (GABA) analogue that is frequently preferred for neuropathic pain. In recent years, pregabalin has been used for perioperative pain control as it can provide effective opioid-sparing analgesia [34]. In a meta-analysis of the effects of pregabalin on PONV, preoperative pregabalin was associated with a significant reduction in PONV compared to placebo [35].

Capsicum plaster at classical Chinese acupoints is an alternative to acupuncture, which has been reported to be an effective method for reducing postoperative pain and PONV when applied to the acupuncture points [36]. Nefopam is a non-opioid, non-steroidal centrally acting analgesic that has been used as an alternative to opioids to control mild to moderate pain [37]. Nefopam was shown to provide similar postoperative analgesia to ketorolac when used as an adjuvant analgesic with fentanyl-based PCA [38]. However, nefopam itself can induce PONV according to some studies [39, 40]. The emetic effect of nefopam could be the main reason for the poor outcome in this review.

According to the fourth consensus guidelines for the management of PONV, anesthetic risk factors of PONV include volatile anesthetics, nitrous oxide, and opioids [41]. Apfel et al. [42] reported that the use of volatile anaesthetics was the strongest risk factor for PONV, but restricted to the early (0–2 h) not the late (2–24 h) postoperative period. However, for patients undergoing maxillofacial surgery, PONV incidence in postoperative 2–24 h is 2.7 times higher than 0–2 h [43]. This may be the reason why propofol had no significant preventive effect in this review. Also, a study by Ichinohe et al. [44] reported that nitrous oxide did not aggravate postoperative emesis after orthognathic surgery. So we speculate that opioids, not volatile anesthetics or nitrous oxide, are the main factor causing PONV following orthognathic surgery.

In the pathogenesis of PONV, the activation of muscarinic acetylcholine receptor plays an important part [45]. Penehyclidine, a new muscarinic antagonist with high selectivity of the M3 receptor, is widely used as premedication to reduce glandular secretion [46]. Not only in orthognathic surgery but another two studies in thyroidectomy and strabismus surgery found that penehyclidine was helpful in preventing PONV [46, 47]. The major concern of penehyclidine is potential cognitive side effect. A meta-analysis found that penehyclidine was not associated with increased incidence of postoperative delirium when compared with either scopolamine or placebo [48].

Bleeding is the second most serious complications of orthognathic surgery, which mainly occurs during down fraction of the maxilla after Le Fort I osteotomy or during separation of the pterygoid junction [49, 50]. BMI, circulating blood volume, nasal mucosal injury, and operative time were associated with the risk of intraoperative massive bleeding in orthognathic surgery [51]. It is widely believed that swallow surgical fluids, specifically blood, during surgical procedures contribute to PONV [52]. This theory is supported by the high incidence of PONV in patients undergoing tonsillectomy and adenoidectomy [53]. To decrease blood ingestion, two strategies are emerging in recent years. One strategy is putting throat packs in the pharyngeal cavity, and another is using a gastric tube to aspirate stomach contents.

With insufficient evidence, throat packs are frequently used for decades to prevent blood and pieces of bone aspiration. The ongoing debate about throat packs is whether they can provide a physical barrier against blood and irrigation fluids and reduce the incidence of PONV. A study by Powell et al. can address this question [27]. According to their study, no difference was found for the gastric contents aspirated by a gastric tube when throat packs were used during surgery. Several complications related to throat packs are concerned by clinicians in recent years. The use of throat packs can lead to postoperative sore throat and dysphagia [54]. Vural et al. [55] used chlorhexidine/benzydamine to soak throat packs and observed reduced postoperative throat pain. However, the incidence of PONV was not statistical different when compared with saline-soaked throat packs. More seriously, if the throat packs were forgot to be removed before tracheal extubation, it would result in airway obstruction and even death [56].

Oliveira et al. [29] reported the beneficial effect of gastric aspiration to prevent PONV, whereas Schmitt et al. [28] did not. The main limitation of Schmitt’s study is the small number of patients (12 patients in each group) and the standardization of the anesthetic protocol. These factors may cause bias and influence the reliability of the result. Furthermore, a retrospective study of Wang et al. [57] analyzed 772 patients to discuss the relationship between gastric negative pressure suction and the incidence of PONV after orthognathic surgery. Their results revealed that the incidence of PONV was halved when patients received gastric negative pressure suction.

The enhanced recovery after surgery (ERAS) is a pathway designed to improve patient outcomes, minimize postoperative complications, and reduce the length of hospital stay [58]. Two retrospective studies by Brookes et al. [59] and Stratton et al. [60] assessed the impact of ERAS protocols on PONV after orthognathic surgery. These studies mainly combined pharmacologic and non-pharmacologic methods such as prophylactic antiemetics, multimodal analgesia, propofol-based TIVA, gastric aspiration at the end of surgery. Both of them reported that the incidence of PONV was significantly decreased by using ERAS protocols.

## Conclusion

Based on current evidence, we conclude that prophylactic antiemetics like dexamethasone, ondansetron, and penehyclidine are the first defense against PONV. Multimodal analgesia with nerve block techniques and non-opioid analgesics should be considered due to their notable opioid-sparing and PONV preventive effect. For the non-pharmacological methods, throat packs are not recommended for routine use because of their poor effect and serious complications. More prospective RCTs are required to confirm whether gastric aspiration can prevent PONV effectively for patients undergoing orthognathic surgery.

## Data Availability

The datasets used during the current study available from the corresponding author on reasonable request.

## Abbreviations

PONV: Postoperative nausea and vomiting
PON: Postoperative nausea
POV: Postoperative vomiting
PRISMA-ScR: Preferred reporting items for systematic reviews and meta-analyses extension for scoping reviews
PCA: Patient-controlled analgesia
PACU: Postanesthesia care unit
GABA: Gama-aminobutyric acid
ERAS: Enhanced recovery after surgery
